# Stress and deformation characteristics of sea ice in a high-resolution, anisotropic sea ice model

**DOI:** 10.1098/rsta.2017.0349

**Published:** 2018-08-20

**Authors:** H. D. B. S. Heorton, D. L. Feltham, M. Tsamados

**Affiliations:** 1 Centre for Polar Observation and Modelling, Department of Meteorology, University of Reading, Reading RG6 6BB, UK; 2 Centre for Polar Observation and Modelling, University College London, London WC1E 6BT, UK

**Keywords:** sea ice, rheology, deformation

## Abstract

The drift and deformation of sea ice floating on the polar oceans is caused by the applied wind and ocean currents. Over ocean basin length scales the internal stresses and boundary conditions of the sea ice pack result in observable deformation patterns. Cracks and leads can be observed in satellite images and within the velocity fields generated from floe tracking. In a climate sea ice model the deformation of sea ice over ocean basin length scales is modelled using a rheology that represents the relationship between stresses and deformation within the sea ice cover. Here we investigate the link between emergent deformation characteristics and the underlying internal sea ice stresses using the Los Alamos numerical sea ice climate model. We have developed an idealized square domain, focusing on the role of sea ice rheologies in producing deformation at spatial resolutions of up to 500 m. We use the elastic anisotropic plastic (EAP) and elastic viscous plastic (EVP) rheologies, comparing their stability, with the EAP rheology producing sharper deformation features than EVP at all space and time resolutions. Sea ice within the domain is forced by idealized winds, allowing for the emergence of five distinct deformation types. Two for a low confinement ratio: convergent and expansive stresses. Two about a critical confinement ratio: isotropic and anisotropic conditions. One for a high confinement ratio and isotropic sea ice. Using the EAP rheology and through the modification of initial conditions and forcing, we show the emergence of the power law of strain rate, in accordance with observations.

This article is part of the theme issue ‘Modelling of sea-ice phenomena’.

## 1. Introduction

Sea ice floating on the polar oceans is composed of many individual floes and floe aggregates. These floes are continually breaking apart, sliding against one another and thermodynamically healing. These interactions result in discontinuous drift and deformation fields. Sea ice is continually under the influence of external wind and ocean forcing that, along with the internal ice stresses resulting from floe interaction and deformation and Coriolis acceleration, make up the sea ice force balance [1,2].

Accurately representing the sea ice force balance is essential for modelling the drift and thickness distribution of sea ice over seasonal and climate time scales. The resulting sea ice drift is important in the momentum transfer from the atmosphere, through sea ice, to the ocean below, driving basin-scale currents in the Arctic Ocean.

The relation between stress and deformation of sea ice is known as sea ice rheology. When modelling the drift and deformation of sea ice within a numerical climate model, a numerical implementation of the rheology of sea ice over ocean dynamical length scales is needed. In recent years, new anisotropic rheologies have been developed that take inspiration from observable oriented features in satellite observations, for example, the elastic anisotropic plastic (EAP) rheology used in this paper [3] and the Maxwell elasto-brittle rheology [4]. The EAP rheology has been shown to produce significantly different sea ice drift and thickness distribution patterns in the Arctic Ocean compared to the isotropic elastic viscous plastic (EVP) rheology [2].

The deformation and force balance of 10 cm samples of sea ice have been investigated in laboratory settings [5]. Typically, these samples are compressed in two dimensions until they fail. The ratio of the magnitude of stress in two dimensions, the stress confinement ratio *R*_int_ (as described in §2a(i)), is found to control the mechanical failure of the ice. For *R*_int_ below a critical confinement ratio *R*_crit_, ice fails with Coulombic lines of shear deformation, lines that are inclined to the principal components of the stress tensor. For *R*_int_ > *R*_crit_ there is a different failure mechanism, with either a single unoriented feature or the disintegration of the sample [6]. These deformation characteristics are argued to be present in sea ice over all length scales [7,8], which is the underlying physical principle behind the EAP rheology presented in §2a(i).

The drift and deformation of sea ice have been observed both from floating arrays of remote buoys [9], and from comparing images from orbiting satellites [10]. The distribution of deformation from these sources is proposed to follow a power law distribution [9,11]. This distribution describes the probability density of the magnitude of strain rate over the entire sea ice pack, with likelihood of a strain rate proportional to that strain rate to a power *r*_c_ < 0. This distribution has been used to investigate how well climate sea ice models represent the deformation and thus the rheology of sea ice over climate length and time scales [12], with higher-resolution models typically better able to reproduce the power law distribution.

In this paper, we document our study into the link between observable deformation features and the underlying internal stress characteristics. We use a state of the art sea ice climate model, the Los Alamos sea ice model (CICE), with the EAP and EVP rheologies on an idealized domain, documented in §2a,b. We use idealized wind forcing in order to impose stress states within the sea ice that produce particular deformation patterns, §2c. We explore the model responses at high resolution, §2d. We explore the link between stress confinement in the wind and internal ice stresses and the resulting deformation patterns, §3b. In the case of EAP, we look at the effect of the anisotropic alignment of the sea ice structure upon deformation characteristics, particularly the deformation characteristics of reorienting winds and sea ice structural alignment, §3c.

## 2. Model configuration

### (a) Model equations

The numerical sea ice model CICE calculates the drift and deformation of sea ice using the vertically integrated, horizontal momentum balance [13]:
2.1
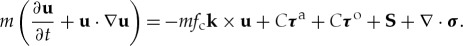
The left-hand side of the equation represents the rate of change of momentum, with **u** the sea ice drift velocity and *m* the mass of sea ice per unit area, balanced by, in order: the Coriolis acceleration −*mf*_c_**k** × **u**, with *f*_c_ the Coriolis parameter and **k** the unit vector normal to the sea surface; the ice concentration *C* weighted applied stress from the atmosphere ***τ***^a^ and ocean ***τ***^o^; gravitational acceleration from the ocean tilt ***S*** set to zero for this study; and the divergence of the internal ice stress tensor ∇ · ***σ***.

The atmospheric drag is calculated from the 10 m wind speed ***U***_a_ with ***τ***^a^ = *ρ*_a_*C*_a_|***U***^a^|***U***^a^, where *ρ*_a_ is the air density and *C*_a_ is a drag parameter. Ocean stresses are typically calculated from the difference between ice and ocean velocities along with a turning angle. In this study, we have a still ocean and no turning angle with ***τ***^o^ = − *ρ*_o_*C*_o_|***u***|***u***|, where *ρ*_o_ is the ocean density and *C*_o_ an ocean drag coefficient.

#### (i) Elastic anisotropic plastic rheology

To represent floe-scale interactions within a continuum model, Wilchinsky & Feltham [3] developed the EAP rheology that sums together the forces arising between many diamond-shaped floes within an arbitrary area of sea ice cover. This rheology was implemented into the Los Alamos sea ice model CICE by Tsamados *et al.* [14], with further investigations into its role in the sea ice force balance presented by Heorton *et al.* [2]. The orientation of ice floes within the area is recorded using a model variable that changes to represent the breaking and healing of ice floes. When considering the interaction between individual floes within an arbitrary area, one is able to derive the plastic sliding and ridging stresses that are combined to give the full local ice stress:
2.2
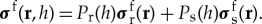
The local sliding, ***σ***^f^_s_, and ridging, ***σ***^f^_r_, stresses are obtained for the floe orientations (given by unit vector ***r***) and their relative motion (determined from strain rate 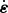) as described by Wilchinsky & Feltham [3]. *P*_(r,s)_(*h*) are the ridging and sliding ice strengths for ice of thickness *h*. The individual floe stresses are expanded over an arbitrary area 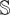 containing many floe alignments represented by *ψ*(***r***) = *ψ*( − ***r***) (with 
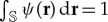
) and total internal stress given by

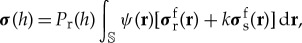
where the sliding ice strength *P*_s_ = *kP*_r_ with *k* a constant.

The structure tensor, used to represent floe orientation, is similarly defined as
2.3
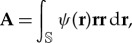
which is a tensor of unit trace, i.e. *A*_11_ + *A*_22_ = 1 with eigenvalues *A*_1_, *A*_2_ = 1 − *A*_1_ associated with eigenvector ***A***_1_, the principal component of ***A*** giving the direction of local anisotropic alignment ***r***. The largest eigenvalue 0.5 < *A*_1_ < 1 indicates the level of local anisotropy, with *A*_1_ = 1 being fully anisotropic and *A*_1_ = 0.5 fully isotropic. The structure tensor changes in time due to local forcing with
2.4
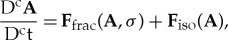
where D^c^/D^c^*t* is the co-rotational time derivative accounting for advection and rigid body rotation and the ***F***_( )_ terms represent various processes that realign ice floes.

***F***_frac_ determines the ice floe reorientation due to fracture, and explicitly depends upon sea ice stress direction but not its magnitude. Following Wilchinsky & Feltham [3], we use four failure modes defined by the internal stress confinement ratio *R*_int_ = *σ*_1_/*σ*_2_, where *σ*_1,2_ are the principal components of the stress tensor: (i) biaxial tension causing longitudinal splitting; (ii) uniaxial compression/tension causing axial splitting; (iii) biaxial compression with a low confinement ratio causing in-plane shear rupture; and (iv) biaxial compression with a large confinement ratio causing out-of-plane shear rupture. Modes (i), (iv) cause no realignment of floes and modes (ii), (iii) align floes with ***r*** parallel to the direction of greatest compressive stress (*σ*_2_). The formulation for ***F***_frac_ is
2.5
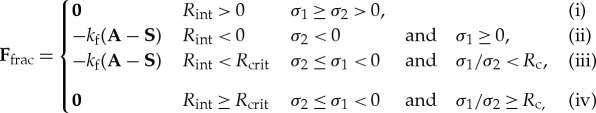
where ***S*** is a tensor that results in the major principal axis of ***A*** aligning with the principal direction of ***σ*** associated with *σ*_1_; *k*_f_ reflects the rate of fracture formation in the sea ice cover and, as with [14], we choose the reference value of *k*_f_ = 10^−3^ s^−1^ which gives 90% anisotropic alignment within 6 h of case (ii) or (iii) occurring. The value of *R*_c_ = 0.3 used is in accordance with the laboratory-scale observations of Golding *et al.* [6] and Iliescu & Schulson [5]. The thermodynamic healing of the sea ice structure tensor is turned off (***F***_iso_ = **0**) to allow us to focus on floe reorientation due to fracture ***F***_frac_.

#### (ii) Elastic viscous plastic rheology

The EVP rheology [15] is a numerical implementation that elastically approximates the viscous plastic (VP) rheology of Hibler [16]. In this rheology, the deformation of the sea ice is modelled as plastic for high stress states and as a highly viscous fluid for low stress states to ease the numerical complexity of distinguishing between plastic and non-plastic deformation (see [13] for further description). This is represented through the stress tensor with
2.6a
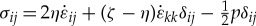
and
2.6b
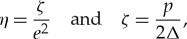
where *η* and *ζ* are the shear and bulk viscosities, *e* defines the elliptical plastic yield curve aspect ratio, 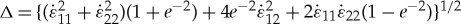 is a scaling factor representing the magnitude of local strain and *p* is the ice strength as discussed below.

### (b) Model domain, boundary and initial conditions

We use a square grid with constant Coriolis acceleration of *f*_c_ = 1.46 × 10^−4^ s^−1^ to make the model applicable to the polar regions. The size of the domain is *d* = 2000 km square. To simplify the model dynamics, we use a single thickness category and the ice strength parametrization of Hibler [16] with 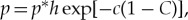 where p* = 2700 N m^−2^ and *c* = 20 are constants. Tests showed that using the alternative strength parametrization of Rothrock [17] and five thickness categories made no qualitative difference to our simulation results. The ice strength parametrization used is the same as that used by Hutchings *et al.* [18], though we have differences in the way the strength and thus internal stresses are initialized, as discussed below.

**Figure 1.**
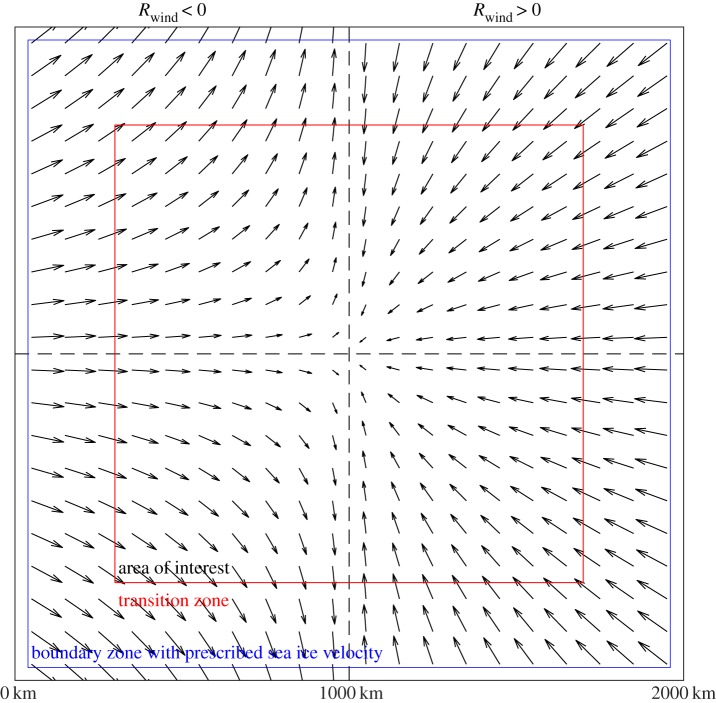
Model domain with two example wind fields. To the left of the figure are flow arrows for a wind with *R*_wind_ =  − 0.8 < 0 and the right of the figure with *R*_wind_ = 0.8 > 0. The black dashed lines represent the lines of reflectional symmetry in the wind forcing field. The blue square shows *d*_bc_, the boundary region with prescribed ice velocity, and the red square shows *d*_trans_, far from the domain boundary within which results are taken from.

To simulate the stress characteristics within the continuous sea ice pack special boundary conditions are needed (figure 1). Boundary conditions for open or closed boundaries cause either too little or too much stress and thus no deformation features, due to dissipation of stress or the ‘locking up’ of the sea ice pack, respectively. For this reason, we use the boundary conditions used in Hutchings *et al.* [18] to produce realistic deformation characteristics on a square grid. For a buffer region of *d*_bc_ ≈ 50 km from the ice edge (tuned for each model resolution to give realistic deformation characteristics, figure 1), we force the sea ice to be in a quasi-free drift state with spatially uniform stress and strain. After stress equations are solved within the elastic sub-cycle (described fully within Hunke *et al.* [19]), giving realistic stress conditions across the whole domain, we stop the internal ice stresses from dissipating out of the domain by imposing the sea ice drift speed within the buffer region (*d*_bc_) by balancing the wind forcing and a linear ocean drag with
2.7
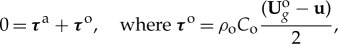
where ***U***^o^_*g*_ = 0 represents the still ocean. As experienced by Hutchings *et al.* [18] this boundary condition gives uniform stress and strain rate within the buffer region as seen in the later figure in §3a. The model simulations presented in this paper are numerically stable throughout. Over a transition region of size *d*_trans_ = 300 km from the domain edge, the uniform strain rate changes into observable features. The inner region beyond *d*_trans_ is the area analysed within the results sections (figure 1).

The model is initialized using data from an Arctic-wide CICE run using the restart configuration. There are many variables required to restart a model run. Most we take from a single point in the Arctic-wide run with 2% noise, with idealized: sea ice velocity near zero, sea ice concentration of 0.999, a constant thin snow cover of 0.1 m and constant idealized sea ice structure tensor and thickness, which we discuss in §3. The rest of the CICE model is unmodified with the thermodynamic and thickness distribution equations solved for. Our model set-up is in contrast to Hutchings *et al.* [18] who solve the VP rheology over a uniform ice cover. So as to allow discrete kinematic features to form, they also introduce noise to the otherwise continuous ice field, but into the initialization of ice strength only.

### (c) Idealized forcing

In order to gain insight into the role of sea ice rheology in producing sea ice deformation features, we perform simulations with idealized atmospheric and oceanic forcing. To induce near constant internal stress conditions over the idealized domain, we impose a wind forcing similar to that used both by Wilchinsky *et al.* [20,21] for discrete element simulations of sea ice and by Hutchings *et al.* [18]. In all model runs, the ocean velocity ***U***^o^_*g*_ is set to zero. We construct wind forcing by imposing the gradient of the wind stress, with *τ*^a^_*yy*_/*τ*^a^_*xx*_ = *R*_wind_ a constant and the maximum wind speed **U**^a^_max_. The wind velocity field can be generated from 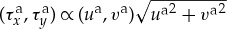 and the boundary conditions of (*u*^a^, *v*^a^) = (**U**^a^_max_, 0)|^*y*=*d*/2^_*x*=0_ and (*u*^a^, *v*^a^) = ( − **U**^a^_max_, 0)|^*y*=*d*/2^_*x*=*d*_, measuring (*x*, *y*) from the southwest corner of the model domain with
2.8
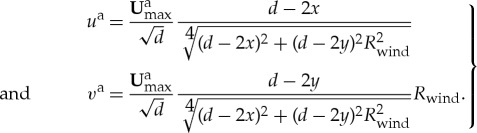
This wind pattern has winds heading eastwards from the western edge of the domain and westwards from the eastern edge of the domain. The winds diverge out of the northern and southern edges for *R*_wind_ < 0 (see the left-hand side of figure 1), and head southwards from the northern edge of the domain and north from the southern edge of the domain for *R*_wind_ > 0 (right-hand side of figure 1). The velocity field is symmetric about the lines *x* = *d*/2 and *y* = *d*/2. The model is initialized with a zero wind field (*u*^a^, *v*^a^) = **0** that increases linearly to the idealized wind forcing set over 6 h of model time.

### (d) Model stability

#### (i) Resolution

**Figure 2.**
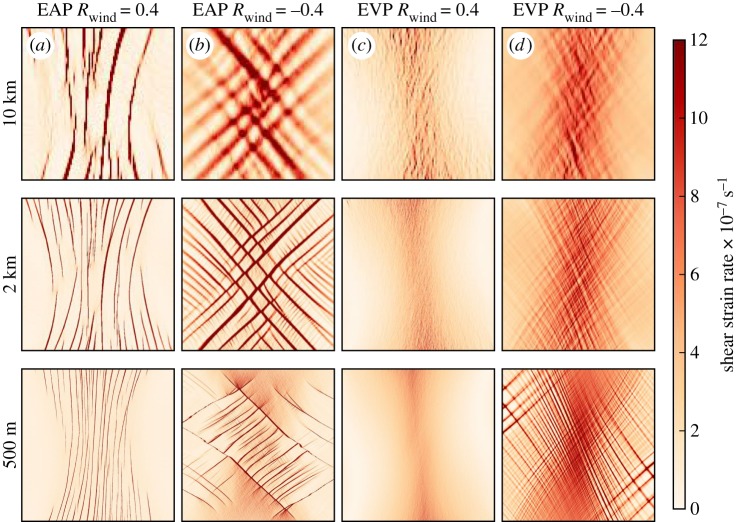
Shear strain rate after 6 h of *R*_wind_ = 0.4 (*a*,*c*) and *R*_wind_ =  − 0.4 (*b*,*d*) winds using the EAP (*a*,*b*) and EVP (*c*,*d*) rheology from an initial uniform unaligned 2 m thick cover of sea ice for increasing resolution as labelled to the left of each row. The area included in the plots is as indicated in the later figure, in §3a, for all the resolutions.

The model resolution has been tested at 10 km, typical of modern high-resolution global climate models, 2 km, typical of very high-resolution regional models, and 500 m. The time resolution is decreased for finer spatial resolution to allow the equations of motion to be solved, with a time step of 600 s at 10 km, 30 s at 2 km and 5 s at 500 m. For all cases, we use 200 elastic sub-cycles within the CICE numerical solver. These time steps give model convergence at each resolution with the model results not changing by more than 1% for shorter time steps or increased sub-cycles. The model responses have been widely tested to confirm a correct response. For example, for the 2 km EVP runs presented in figure 2, the same result is achieved for a model time step of 5 s and 1000 sub-cycles.

The deformation patterns for the EAP rheology in figure 2 show good correlation for increasing resolution. The linear deformation features have a similar shape for the three resolutions. At increasing resolution, finer details can be observed: finer parallel slip lines for *R*_wind_ = 0.4 and the appearance of comb cracks at 2 km and 500 m resolution for *R*_wind_ =  − 0.4. When using the EVP rheology (figure 2) only the 500 m resolution under winds with *R*_wind_ =  − 0.4 gives linear deformation features after only 6 h of model time.

We have repeated many of the runs presented in this paper with the wind field rotated by 45° about the centre of the domain to test the numerical sensitivity of our chosen square grid. The characteristic lines of deformation and the principal component of the structure tensor rotate with the forcing field as seen in the rotating wind experiments in the penultimate figure in this paper.

#### (ii) Model run length

**Figure 3.**
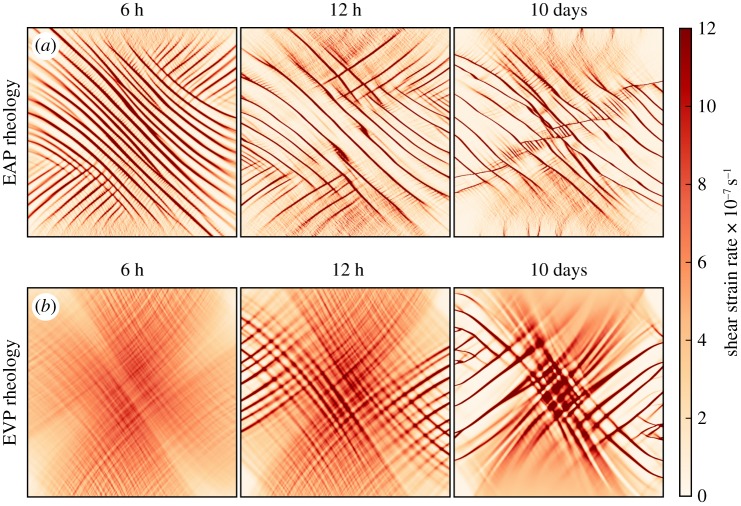
Shear strain rate over 10 days of winds with *R*_wind_ =  − 0.8 in using the EAP rheology (*a*) and EVP rheology (*b*). For the EAP rheology, the deformation patterns are similar from 12 h to the end of the run (10 days). The EVP rheology results in fewer strong linear features and they take longer to appear ( ≈ 3 days).

The sea ice model has been run for 10 days of model time at 2 km resolution under winds with *R*_wind_ =  − 0.8 (shown in figure 3), chosen to produce high rates of deformation and minimal mechanical thickening at the centre of the domain. We test whether the emergent deformation characteristics are stable or change with time.

For the EAP rheology oriented lines of deformation are observable from 3 h of model time, and the sea ice is strongly aligned (*A*_1_ > 0.95) from after 10 h of model time. From 12 h to 10 days, the features have little variation. All differences between these two time points are due to thickening sea ice, primarily in areas of ridging.

With the EVP rheology, the model takes longer to stabilize. There are no linear deformation features at 6 h and few at 12 h. Identifiable evenly spaced lines of deformation are apparent from 3 days of model run and remain until the end of the run. Using a higher resolution may give more identifiable features (see figure 2), but is computationally beyond the scope of this study.

## 3. Results

### (a) Reference runs with the EAP rheology, initially isotropic

**Figure 4.**
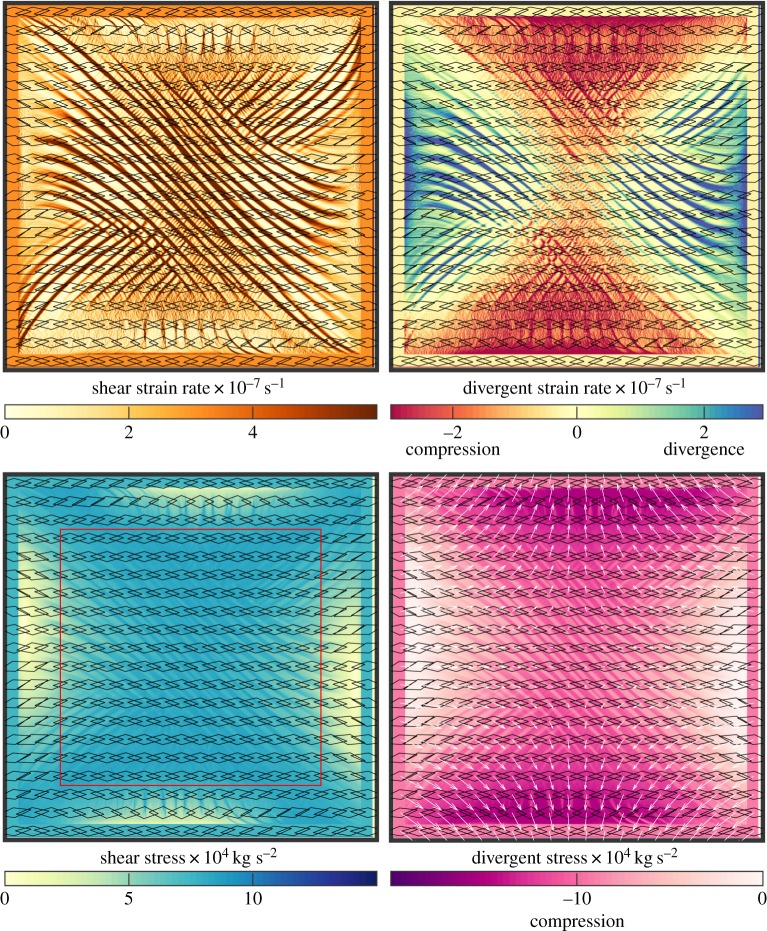
Sea ice deformation and stress after 6 h of *R*_wind_ =  − 0.8 winds from an initial uniform unaligned 2 m thick cover of sea ice using the 2 km resolution. Shear and divergent components of the sea ice strain rate and internal stress tensors with the anisotropic structure tensor displayed by the overlaid quadrilaterals with a diamond shape indicating high anisotropic alignment. The whole domain is displayed with the outer 100 km boundary conditions clearly visible. The red square in the shear stress plot shows the area plotted in figures 2–6.

**Figure 5.**
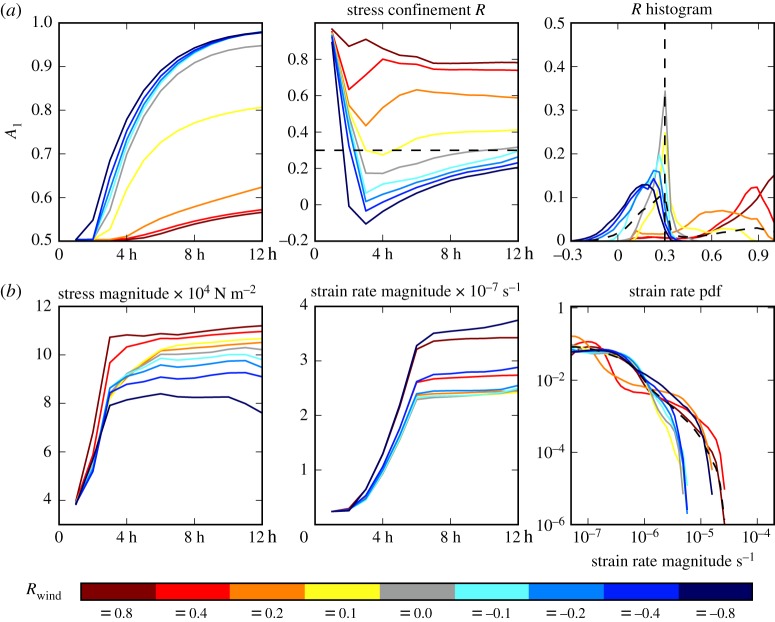
Sea ice conditions from an initial uniform unaligned 2 m thick cover of sea ice using the 2 km resolution and varying wind stress confinement *R*_wind_ shown in the legend at the bottom. (*a*) The developing average alignment *A*_1_ and internal stress confinement *R* and the histogram of *R* from 4 h when the stress state stabilizes. (*b*) The average of stress and strain rate magnitudes and the pdf of the strain rate magnitude from the whole run. Values are taken from within the red square shown in figure 4.

Here we present the stress–strain rate relationship for two reference runs with an initial isotropic sea ice cover (*A*_1_ = 0.5): the case of *R*_wind_ =  − 0.8 and divergent shear deformation (figure 4) and of *R*_wind_ = 0.8, both shown in figure 5. The wind speed increases linearly over the first 6 h of model run, with the wind stress and total strain rate being proportional to the square of the wind speed; see dark red and dark blue lines in figure 5. The internal stress magnitude increases to a steady value by 3 h of model run. The internal stress confinement *R*_int_ remains near constant for *R*_wind_ = 0.8 from 3 h onwards. For *R*_wind_ =  − 0.8, *R*_int_ is increasing from 3 h to the end of the run as the sea ice becomes increasingly anisotropically aligned (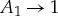).

In figure 4 we show the shear and divergent strain rate and internal stress for the run with *R*_wind_ =  − 0.8. The internal stresses are near spatially uniform with lines of reduction in stress corresponding to lines of high deformation, and reduced or increased stress near the domain boundaries. The lines of deformation have high shear strain rate and either divergent or compressive failure. For all the runs with higher *R*_wind_, divergent deformation is rare and the lines of shear only correspond to compressive failure. In the centre of the domain, far enough from the imposed boundary conditions, there is near uniform stress and the lines of deformation are evenly spaced. The confinement of the internal stress is less than the critical confinement ratio *R*_int_ < *R*_crit_ for the whole run, causing the sea ice structure to become anisotropically aligned as indicated by the overlaying quadrilaterals in figure 4 being diamond shaped. The principal component of the sea ice structure tensor (***A***_1_, as indicated by the major axis of the diamonds) is aligned to the principal component of the wind stress gradient, not the wind direction. This is in agreement with Heorton *et al.* [2] who found highly variable angles between the direction of wind stress and sea ice structure within the central Arctic.

**Figure 6.**
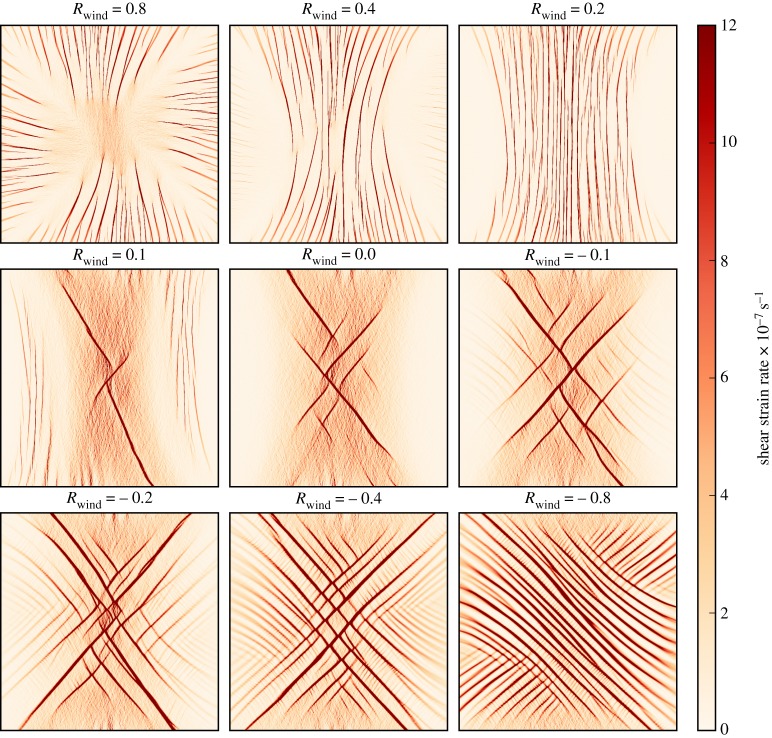
Shear strain rate after 6 h of winds from an initial uniform unaligned 2 m thick cover of sea ice using the 2 km resolution. The wind stress confinement *R*_wind_ is varied from 0.8 to −0.8 as labelled above each plot. The area included in the plots is as indicated in figure 4.

For the convergent wind field with *R*_wind_ = 0.8 shown in figure 6 the compressive stress and strain rate dominates. The centre of the domain is dominated by a region of near constant compressive stress and deformation with little shear stress. Radiating away from the central region there are slip lines of shear and compressive deformation that correspond to reduced compressive and increased shear stresses. The majority of the sea ice remains isotropic, with the sea ice within slip lines becoming anisotropically aligned (not shown).

### (b) Varying the internal stress confinement *R*_int_, initially isotropic

Here we investigate the relationship between the internal stress confinement ratio *R*_int_ and the deformation characteristics of sea ice displayed in figures 6 and 5. Since *R*_int_ is an emergent property we cannot directly impose it and so we perform a set of simulations with different values of imposed *R*_wind_, which directly determines *R*_int_. We calculate *R*_int_ = *σ*_1_/*σ*_2_ from the principal components or eigenvectors of the internal ice stress tensor *σ* for each grid cell point, with the values plotted in figure 5 taken from within the inner region *d*_trans_ from the domain edge.

#### (i) Stress and strain characteristics

As with the runs presented in §3a, the wind speed increases to **U**^a^_max_ = 15 m s^−1^ over the first 6 h of the model run. The magnitude of internal stress reaches its maximum from 3 h of model run whereas the strain rate increases with the wind speed to its maximum at 6 h of model run (see figure 5). The runs with higher *R*_wind_ have greater magnitude of internal stress due to greater confinement. The magnitude of strain rate appears to be equal for the wind confinements *R*_wind_ of equal magnitude (+0.8, − 0.8, for example), due to the symmetry of equations (2.8), resulting in the total applied wind stress for these runs being equal.

In the EAP rheology, the runs that have *R*_int_ < *R*_crit_ become anisotropically aligned (see the *A*_1_ and *R*_int_ panels of figure 5). For increasing alignment within the sea ice cover *R*_int_ coverges to *R*_crit_ = 0.3.

The experiments with *R*_wind_ < 0.1 have curved lines for the probability density function (pdf) of shear strain rate (figure 5), indicating the deformation taking place over many low strain rate events. For *R*_int_ > *R*_crit_ there is a complex curve showing the deformation being collected into fewer, high strain rate features. The combined pdf for all runs (black dashed line) approaches a linear relationship and thus the power law of sea ice deformation suggested by Girard *et al.* [12] and Rampal *et al.* [9].

#### (ii) Emergent failure modes

For an initial isotropic cover of sea ice, varying *R*_wind_ reveals two failure modes of sea ice. The runs with a large positive *R*_wind_ in the range 0.1 < *R*_wind_ < 0.8 produce shear strain rate concentrated into near parallel lines that diverge towards the edge of the domain; see figure 6. The high shear deformation coincides with convergent deformation. The lines are perpendicular to the principal component of the wind stress gradient. This mode we attribute to the out-of-plane shear failure of sea ice, ridging for example, that inspired failure mode (iv) in equation (2.5).

The second failure mode happens for *R*_wind_ < 0.1 and features diagonally intersecting lines of high shear strain rate. The number of lines and incident angle between them both appear to increase for decreasing *R*_wind_; for example the run with *R*_wind_ =  − 0.8 has the greatest number of intersecting lines of shear that intersect at an angle of ≈ 90°. Hutchings *et al.* [18], who performed a fuller analysis of linear feature intersection, also found the same relationship of increasing feature intersection angle for decreasing *R*_wind_. We attribute this failure mode to in-plane shear rupture with the sea ice pack, the inspiration for mode (iii) in equation (2.5). Although mode (ii) can also cause the anisotropic alignment observable in this case, uniaxial compression represented by a negative confinement ratio is little represented within the histogram of confinement in figure 5.

The wind stress confinement *R*_wind_ is not the same as the sea ice internal stress confinement below it. Figure 5 shows an emergent bimodal distribution of *R*_int_. For *R*_wind_ ≤ 0.0, *R*_int_ peaks near, but strictly less than, *R*_crit_. For *R*_wind_ > 0.1 and increasing, *R*_int_ has a peak that approaches 1.0. The peak at *R*_crit_ corresponds to observable Coulombic lines of shear and anisotropy, and the peak at *R*_int_ = 1.0 corresponds to parallel lines of shear and isotropy. The experiment with *R*_wind_ = 0.1 (yellow line) displays both peaks, as the domain is split into isotropic regions with parallel deformation features and anisotropic regions with Coulombic deformation features, as seen in figure 6. The bimodal deformation modes of sea ice have been observed in laboratory experiments. Golding *et al.* [6], for example, found a clear bimodal distribution of slip line intersection angle about *R*_crit_ when performing laboratory experiments with imposed stress confinement. A laboratory experiment where the internal stresses could be observed in comparison to imposed external stresses, if possible, would produce a distribution of stress confinement ratio that can be used to contrast with those we show for the EAP and EVP rheologies in this paper.

### (c) The role of alignment and realignment

When the sea ice internal stress confinement is less than the critical confinement ratio *R*_int_ < *R*_crit_, the sea ice structure tensor becomes aligned (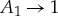). The internal stress characteristics and link between the confinement of wind stress *R*_wind_ and internal stress *R*_int_ are a function of the directional alignment of the structure tensor (the direction of ***A***_1_). To investigate these relationships we perform the runs described above in §3b but with a pre-aligned sea ice structure tensor and contrast with the EVP rheology. To link the different sea ice structures together, we investigate longer runs with changing wind patterns.

#### (i) Pre-aligned initial conditions

**Figure 7.**
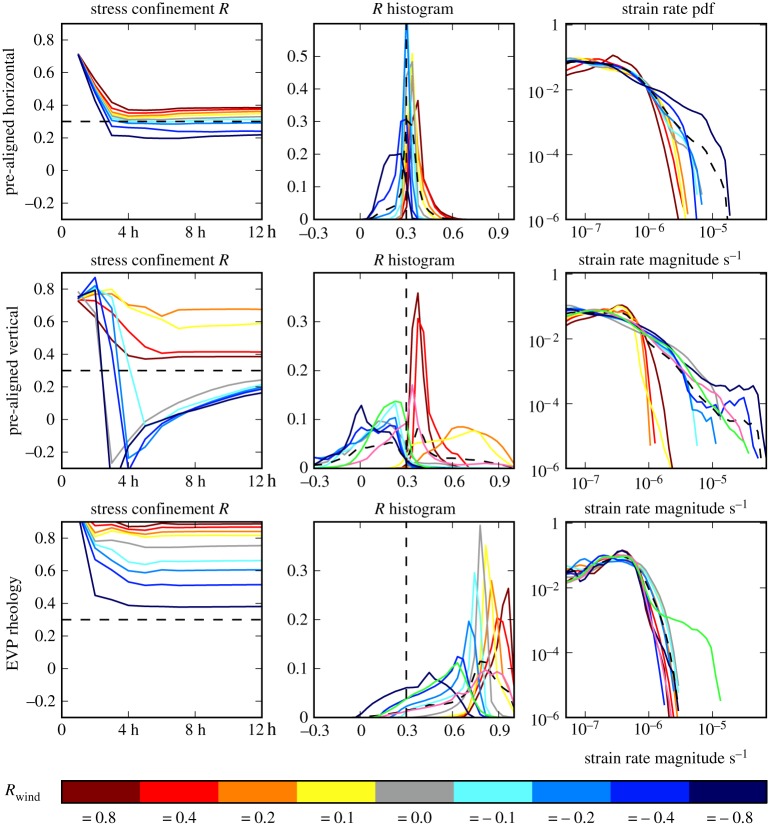
Stress and strain characteristics for pre-aligned sea ice using the EAP rheology and for the EVP rheology using the 2 km resolution. The data presented are from two sets of experiments that are similar to those presented in figures 5 and 6 with altered initial conditions. (*a*) Sea ice pre-aligned with a structure tensor representing diamond shaped floes horizontal, similar to those indicated in figure 4. (*b*) Sea ice pre-aligned with a structure tensor representing diamond shaped floes vertical, perpendicular to those in the top row; these runs feature the rapid realignment of the sea ice internal structure. (*c*) For the EVP rheology that does not consider the alignment of sea ice. The leftmost plots are the time evolution of the average and the centre plots the histograms of the internal stress confinement *R*_int_. The rightmost plots are the pdf of the strain rate magnitude. Extra pink and green lines for the alternating and rotating runs in figure 8, respectively, are included within the *R*_int_ histogram and strain rate pdf in panel (*b*) (EAP rheology) and panel (*c*) (EVP rheology).

For anisotropically aligned sea ice, pre-aligned to the expected alignment for the given wind field (principal component ***A***_1_ aligned with the *x* axis), the parallel slip line failure mode for *R*_int_ > *R*_crit_ shown in figure 6 does not occur. The intersecting Coulombic deformation patterns as shown in figure 6 do occur for *R*_int_ < *R*_crit_ but the sea ice internal stress confinement ratio *R*_int_ is much more closely concentrated around the critical confinement ratio *R*_crit_ for all the runs to produce figure 7. This causes the Coulombic slip lines to only occur for *R*_wind_ ≤ 0.2 for this pre-alignment. For *R*_int_ > *R*_crit_ (for winds with *R*_wind_≥0.2 in this case) the increased shear stress of aligned sea ice and the converging sea ice combine, resulting in little shear deformation and no identifiable features. For the case of *R*_int_ ≈ *R*_crit_ a new deformation pattern is observed as shown in the last figure in this paper. This failure mode has bands of shear failure that are similarly oriented to the Coulombic slip lines but are much wider and have a blurry appearance. In some cases the bands may be formed of closely packed comb cracks perpendicular to the overall band of shear, though more investigation is needed to confirm this. This failure mode is also observed for the *R*_wind_ = 0.0 run in figure 6 if continued for 2 days, at which point *A*_1_ ≈ 1 and conditions are very similar to the pre-aligned case. Apart from the visual features this aligned failure mode has similar characteristics to the Coulombic failure mode: high shear stress, *R*_int_ ≈ *R*_crit_ and a similar pdf of strain rate magnitude.

When the model is initiated with sea ice aligned perpendicular to the expected alignment for the applied wind field (principal component ***A***_1_ aligned with the *y* axis), then there are two failure modes encountered. For *R*_wind_ > 0.0 the sea ice remains anisotropic as in the initial conditions and the increased shear stresses compared to the isotropic case result in no obvious deformation characteristics. For these cases (‘warm’ coloured lines in figure 7*c*), *R*_int_ remains closer to *R*_crit_ than in the isotropic case, with increasing *R*_wind_ resulting in decreased *R*_int_. For *R*_wind_ ≤ 0.0, the sea ice is able to realign with *R*_int_ going lower than *R*_crit_ within the run time. These runs all go through an ≈ 6 h period of realignment where the structure tensor ***A*** and internal stress confinement change rapidly. During this period and afterwards, Coulombic slip lines occur though they move orientation and location much more than in the runs illustrated in figure 6. As the internal stress state changes, divergent weakening occurs, giving the high shear strain rates in the second peak on the pdf in figure 7. For the two failure modes in this experiment there is a bimodal distribution in the histogram for *R*_int_. Those that realign appear to have strictly *R*_int_ < *R*_crit_ and likewise those that do not realign have *R*_int_ > *R*_crit_ for all cases.

In comparison to these runs, when using EVP rheology there is no emergent relationship between *R*_int_ and any critical confinement ratio. There is a different peak in the histogram of *R*_int_ in figure 7 for each experiment with varying *R*_wind_. The pdf of strain rate follows the same curved profile for each EVP experiment.

#### (ii) Changing wind fields: alternating the wind stress confinement *R*_wind_

**Figure 8.**
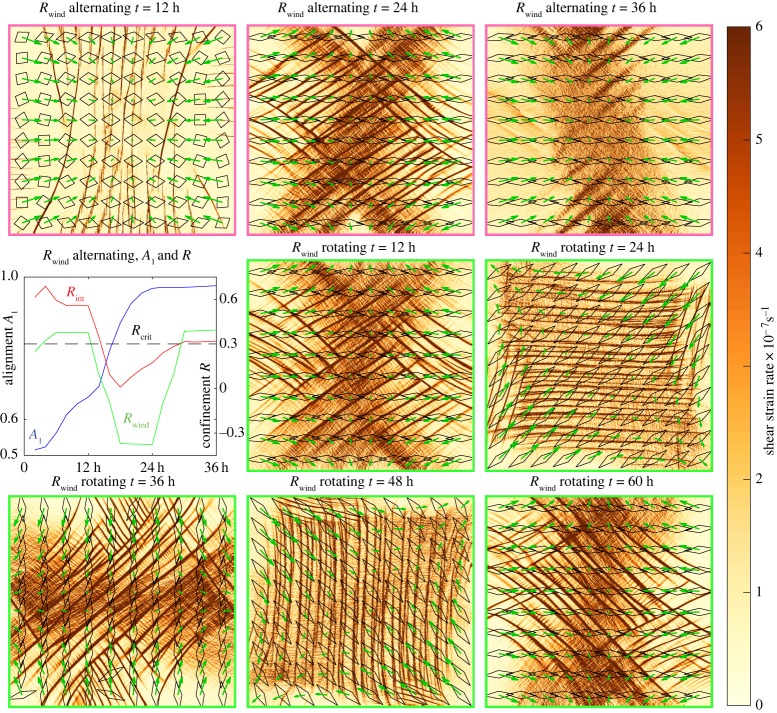
Sea shear strain rate for runs with changing wind conditions with local sea ice structure as indicated by the overlaying quadrilaterals and forcing wind field as shown by the green arrows. The three frames outlined in pink and the line plot are for a run with winds that alternate through *R*_wind_ = (0.4,  − 0.4, 0.4) every 12 h as seen in the green overlaying arrows and the green line. The red line shows the sea ice internal stress confinement *R*_int_ and the blue line the average level of anisotropy *A*_1_. The plots outlined in green are for winds of *R*_wind_ =  − 0.4 that rotate around the centre of the grid by 45° anticlockwise every 12 h as seen in the green overlaying arrows. The changing wind fields are created by setting the winds every 6 h and allowing the model to interpolate in-between. The colour of the plot borders corresponds to the colour of the extra lines on the cumulative strain plots in figure 7.

**Figure 9.**
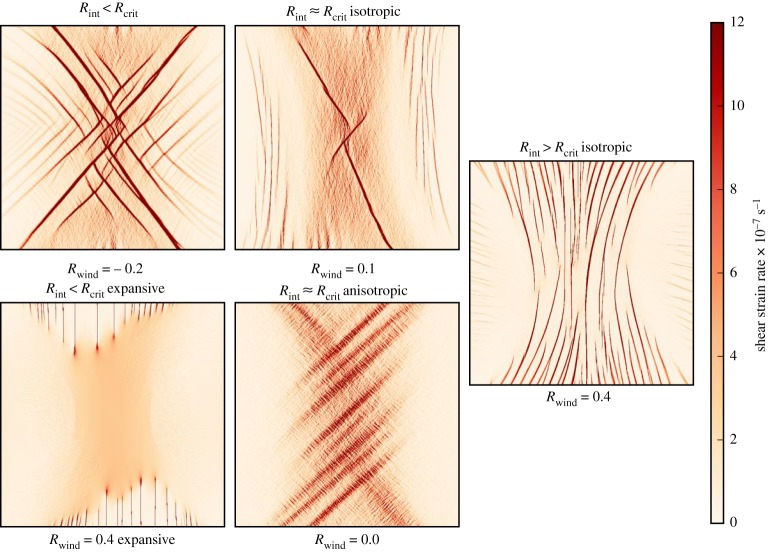
The five distinctive failure modes of sea ice. The shear strain rate after 6 h of model run showing the characteristic deformation of sea ice with internal stress confinement *R*_int_ about the critical confinement ratio *R*_crit_ and for expansive wind stress. The confinement of wind stress *R*_wind_ used for each run is displayed below each figure.

The top three panels edged in pink and the line plot within figure 8 illustrate a simulation where the wind forcing alternates. The wind speed increases linearly from still (*u*^a^, *v*^a^ = 0) conditions at *t* = 0 to a wind field with **U**^a^_max_ = 15 m s^−1^ and *R*_wind_ = 0.4 at *t* = 6 h with the wind field then held constant until *t* = 12 h. From *t* = 12 to 18 h, the wind pattern is smoothly varied to *R*_wind_ =  − 0.4, with the wind speed held constant. From *t* = 24 to 30 h, the wind field is interpolated back to have *R*_wind_ = 0.4 and then held constant until *t* = 36 h, as shown in the line plot in figure 8. The first wind condition causes the isotropic parallel slip lines as found in the *R*_wind_ = 0.4 run in figure 6 with the majority of the ice remaining isotropic and *R*_int_ > *R*_crit_ (blue and red lines in figure 8). As the wind field changes to have *R*_wind_ < 0, the internal stress changes to have *R*_int_ < *R*_crit_ and the sea ice begins to anisotropically align with intersecting slip lines forming as seen in the *R*_wind_ =  − 0.4 run in figure 6. As the sea ice becomes anisotropically aligned, *R*_int_ approaches *R*_crit_, where it remains even after the wind field returns to have *R*_wind_ = 0.4, as the the stress state within the sea ice cannot return the sea ice to isotropy. The deformation field no longer has parallel slip lines and bears a resemblance to the *R*_int_ ≈ *R*_crit_ anisotropic mode in figure 9. The *R*_wind_ =  − 0.4 wind field causes heterogeneous irreversible change to the sea ice alignment.

#### (iii) Changing wind fields: rotating the wind direction

The final five panels edged in green in figure 8 show snapshots of a rotating run where the wind field with **U**^a^_max_ = 15 m s^−1^ and *R*_wind_ =  − 0.4 rotates by 45° anticlockwise about the centre of the grid during *t* = 12–18, 24–30, 36–42 and 48–54 h to return to the wind conditions at 6 h, as this wind field has horizontal and vertical symmetry (see green arrows on figure 8). For each forcing arrangement, intersecting Coulombic shear slip lines occur diagonal to, and the sea ice structure becomes aligned parallel to, the principal component of the wind stress gradient at that point in time (see deformation patterns and overlaying quadrilaterals in figure 8). During the transition between the forcing arrangements, large slip lines occur (see animation in the electronic supplementary material) that are more widely spaced and have higher local strain rates. These higher strain rates occur due to the reorientation of the sea ice structure tensor and result in the linear profile in pdf of strain rate magnitude (see green line in the plot for pre-aligned vertical structure in figure 7). In comparison the pdf for the alternating run also shows some linearity, and the rotating run with the EVP rheology has increased high strain rates compared with the runs with constant wind forcing (green line in the bottom row of figure 7).

## 4. Concluding remarks

We have performed idealized numerical experiments using a sea ice model to investigate the link between applied wind and internal sea ice stress conditions and observable deformation characteristics. We have used a sea ice model commonly used within global climate models (the Los Alamos sea ice model, CICE) with minimal adaptation so it runs with idealized initial conditions on a square grid. We run the model at 10 km, 2 km and 500 m spatial resolutions using both the anisotropic EAP and isotropic EVP rheologies. This set-up enables us to illustrate the sea ice dynamical phenomena that can be expected to occur within state of the art high-resolution sea ice climate models and is suitable for testing and comparing all sea ice model rheologies.

We have successfully imposed internal sea ice stress states using winds fields with a constant confinement of the wind stress gradient (*R*_wind_, see §2c). We have discovered an emergent bimodal relationship between *R*_wind_ and the internal stress confinement *R*_int_. This result links the EAP rheology and laboratory experiments such as Golding *et al.* [6] where a bimodal relationship between imposed stress confinement and fracture alignment is observed, thus successfully continuing the hierarchy of ice failure over all length scales [7]. With the anisotropic EAP rheology, our numerical experiments with varying *R*_wind_ and initial alignment have revealed five characteristic failure modes as illustrated in figure 9. For *R*_int_ > *R*_crit_, there are two failure modes. When the sea ice is isotropic, parallel shear slip lines can form. However, when the sea ice is anisotropic, its increased shear strength resists the formation of shear slip lines and the sea ice deforms compressively over large length scales. For *R*_int_ < *R*_crit_, the sea ice becomes anisotropically aligned due to the mechanics of the rheology in equation (2.5) and there is only one distinctive failure mode, intersecting shear slip lines. A further failure mode occurs for diverging sea ice, where *R*_wind_ = 0.4 with the wind on a bearing from the centre to the edge of the domain. In this case, the sea ice fails in divergence, *R*_int_ < 0 and the sea ice becomes anisotropically aligned.

Previous attempts at characterizing the rheology of sea ice over basin length scales have focused on the distribution of strain rate [11], observable from satellite observation of sea ice drift and an emergent property of sea ice models [12]. Observations show that the pdf of strain magnitude follows a power law when collecting the data from over basin and seasonal length and time scales [9]. In this paper, we investigate the distribution of strain rate for constant and changing stress conditions. We discover that both the EAP and EVP have fewer high strain rate events compared to the expected power law when considering an individual model run with constant stress conditions. However, when using the EAP rheology and either forcing the sea ice structure to rapidly realign or using changing wind conditions, a power law emerges (see figure 7, discussed in §3c). When considering previous studies into the time scaling of sea ice deformation [22–24], this behaviour is expected. This result suggests that for uniform wind conditions, calm periods between Arctic winter storms for example, the observed pdf of strain rate magnitude may not follow a power law. However, due to the scale of the Arctic and that the deformation of sea ice results from internal stresses, including at its boundaries, the likelihood of low stress conditions throughout the Arctic may be low.

In this paper, we have shown clear contrasting features of the EAP and EVP rheologies when using them as part of the currently available CICE model set-up. The EAP rheology consistently gives observable deformation features for all the model resolutions studied, but only for specific situations when using the EVP rheology (§2d). The EAP is also able to produce a power law for the probability of strain rate magnitude and has an emergent critical confinement ratio for the internal sea ice stresses (figure 8).

## Supplementary Material

Animated version of figure 8

## Data accessibility

This article has no additional data.
