# Supplementary figures and images for: Stress and deformation characteristics of sea ice in a high-resolution, anisotropic sea ice model
Source: Philos Trans A Math Phys Eng Sci. 2018 Aug 20;376(2129):20170349. doi: 10.1098/rsta.2017.0349 (PMC6107622; doi:10.1098/rsta.2017.0349)

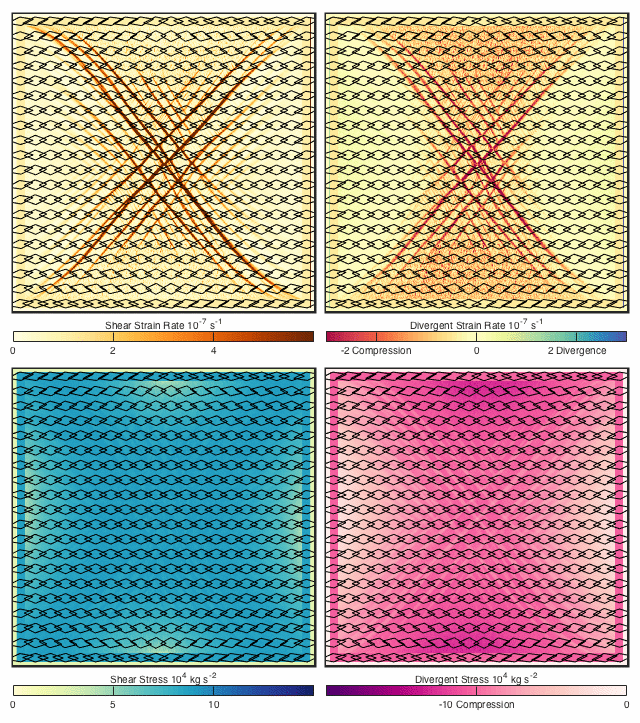

Supplement: Animated version of figure 8 [file rsta20170349supp1.gif]
